# Effect of Curing Conditions on the Hydration of MgO in Cement Paste Mixed with MgO Expansive Agent

**DOI:** 10.3390/ma16114032

**Published:** 2023-05-28

**Authors:** Xuefeng Zhao, Zhongyang Mao, Xiaojun Huang, Penghui Luo, Min Deng, Mingshu Tang

**Affiliations:** 1 College of Materials Science and Engineering, Nanjing Tech University, Nanjing 211800, China5967@njtech.edu.cn (X.H.); 202162103025@njtech.edu.cn (P.L.);; 2 State Key Laboratory of Materials-Oriented Chemical Engineering, Nanjing 211800, China

**Keywords:** MgO expansive agent, variable temperature curing, hydration degree, curing condition

## Abstract

Using the volume expansion generated by the hydration of the MgO expansive agent to compensate for the shrinkage deformation of concrete is considered to be an effective measure to prevent concrete shrinkage and cracking. Existing studies have mainly focused on the effect of the MgO expansive agent on the deformation of concrete under constant temperature conditions, but mass concrete in practical engineering experiences a temperature change process. Obviously, the experience obtained under constant temperature conditions makes it difficult to accurately guide the selection of the MgO expansive agent under actual engineering conditions. Based on the C50 concrete project, this paper mainly investigates the effect of curing conditions on the hydration of MgO in cement paste under actual variable temperature conditions by simulating the actual temperature change course of C50 concrete so as to provide a reference for the selection of the MgO expansive agent in engineering practice. The results show that temperature was the main factor affecting the hydration of MgO under variable temperature curing conditions, and the increase in the temperature could obviously promote the hydration of MgO in cement paste, while the change in the curing methods and cementitious system had an effect on the hydration of MgO, though this effect was not obvious.

## 1. Introduction

Although concrete has been used as a building material for a long time, some basic problems have not been completely solved. The shrinkage deformation of concrete leading to the decline of durability is one of the most important problems [1,2,3,4]. The shrinkage deformation of concrete is a non-external deformation caused by the combined action of physics and chemistry. In actual structures, this volume deformation is often limited by external constraints, resulting in stresses within the concrete [5]. The cracking of concrete not only reduces the mechanical properties of the structure and affects the beauty of the concrete building but also provides a convenient channel for harmful ions to enter the concrete interior, which could further affect the durability of the concrete and reduce the service life of the concrete structures [6]. When the durability of the concrete decreased seriously, a large amount of production materials was spent on either the maintenance or even the reconstruction of the concrete structure, which is contrary to the concept of energy conservation and the emissions reduction proposed today.

In order to reduce the negative effects caused by the shrinkage cracking of concrete, researchers have attempted to use a variety of anti-cracking measures in engineering practice and achieved better anti-cracking effects. The anti-cracking measures for concrete mainly were considered in the following three directions: (1) Reduce the shrinkage deformation generated by the concrete itself: For example, covering the surface of concrete in a timely manner after it has been formed and spraying water on the surface periodically as a means of maintaining surface moisture to reduce the risk of surface cracking [7]; reducing the peak temperature of concrete which decreases the impact of the temperature drop shrinkage (for mass concrete, the value of the temperature rise of concrete can be more than 40 °C, plus the initial temperature value of concrete can be up to 75 °C~85 °C, so the concrete produces a huge shrinkage stress in the cooling stage). The means of reducing the peak temperature of concrete include embedding cooling water pipes inside the mass concrete, taking away part of the heat by cooling circulating water [8,9], using low and medium-heat Portland cement to reduce the heat release of cement hydration, replacing part of the cement with mineral admixtures such as fly ash to reduce the heat of hydration of cementitious [10,11], etc. The use of shrinkage reducers to reduce the tension of the internal capillaries of concrete can provide a means of reducing shrinkage due to a lower relative humidity inside the concrete [12,13,14]. The use of internal curing agents (water-saturated low-density aggregates and highly absorbent polymeric materials, etc.) to slow down the drying shrinkage during the hydration of cement by releasing water stored inside the curing agent [15,16,17], etc. (2) Improving the cracking resistance of concrete by adding fibers to concrete increases its tensile strength by virtue of the tensile capacity of the incorporated fibers and inhibits the possibility of internal microscopic cracks that can develop into macroscopic cracks [18,19,20,21]. (3) Adding an expansion component to concrete by adding expansion sources to concrete can compensate for the shrinkage deformation of concrete through the volume expansion generated by the expansion component during hydration, thereby alleviating or avoiding the generation of shrinkage cracks [22,23,24].

The above anti-cracking means have proved their effectiveness through long-term practice, but the shortcomings of some anti-cracking measures have also been found in the process of this practice. For the mitigation of the thermal shrinkage of mass concrete, the use of the cooling water pipe can play a role in reducing thermal shrinkage, but there are problems, such as complex construction, the high cost of cooling facilities and a long construction cycle [8,25]. In addition, adding fiber to concrete can enhance the tensile properties of the concrete, but there are also some problems such as the addition of fiber which leads to the poor fluidity and workability of concrete. Additionally, how to make the fiber uniform distribution in the concrete is also a problem to be solved [26]. Relatively speaking, adding an expansive agent to concrete is an economical, simple and effective control method [23].

At present, the common expansive agent mainly includes a sulfur aluminate type expansive agent, calcium oxide expansive agent and magnesium oxide expansive agent. The sulfur aluminate expansive agent hydration requires a large amount of water, and the thermal stability of sulfur aluminate hydration products is poor, meaning it can undergo dehydration decomposition at temperatures higher than 70 °C [24,27]. The hydration rate of the expansion of the calcium oxide expansive agent is very fast, and it is not easy to control [8,28]. Compared to calcium oxide and sulfur aluminate type expansive agents, the hydration expansion of the MgO expansive agent requires less water, and the physical and chemical properties of hydration products are more stable. Moreover, the expansion rate of magnesium oxide can also be regulated and designed, which makes the application of the MgO expansive agent in engineering practice more and more common [29,30].

Although the MgO expansive agent has a good effect on preventing concrete cracking, existing studies mainly focus on the influence of the MgO expansive agent on concrete deformation under constant temperature conditions, while engineering mass concrete experiences a variable temperature process, especially for high-strength concrete, due to a large amount of cementitious material, with higher temperature rises. In order to provide some guidance for the selection of the MgO expansive agent under actual engineering conditions based on C50 concrete engineering, this paper mainly studies the effect of curing conditions on MgO hydration in a cement paste-mixed MgO expansive agent under the actual variable temperature condition. In addition, the hydration of MgO in cement paste formed with two kinds of cementitious systems that are commonly used in engineering was compared.

## 2. Materials and Methods

### 2.1. Materials

The raw materials used in this experiment included Portland cement, secondary fly ash, S95 slag powder and the MgO expansive agent (i.e., MEA). Among them, Portland cement was P·Ⅱ52.5 cement produced by Jiangnan Onoda Cement Co., Ltd., Nanjing, China, and secondary fly ash and S95 mineral powder was provided by Nanjing Pudi Concrete Company, Nanjing, China. Four kinds of MgO expansive agents were provided by Wuhan Sanyuan Special Building Materials Company and Jiangsu Sobute Company. According to the citric acid method [31], the reactivity values of four kinds of MgO expansive agent were 120s, 180s, 240s and 330s. Additionally, they were named MEA-120, MEA-180, MEA-240 and MEA-330, respectively. Table 1 shows the chemical composition of Portland cement, secondary fly ash, S95 slag powder and the four kinds of magnesium oxide expansive agent used in the experiment.

### 2.2. Methods

#### 2.2.1. Simulation of the Temperature Change Process of Mass Concrete

In this experiment, the simulation of the temperature change process of mass concrete was based on the actually measured temperature data of C50 mass concrete in engineering, and the curing box and external temperature acquisition module were used to realize the stage temperature change.

**Figure 1:**
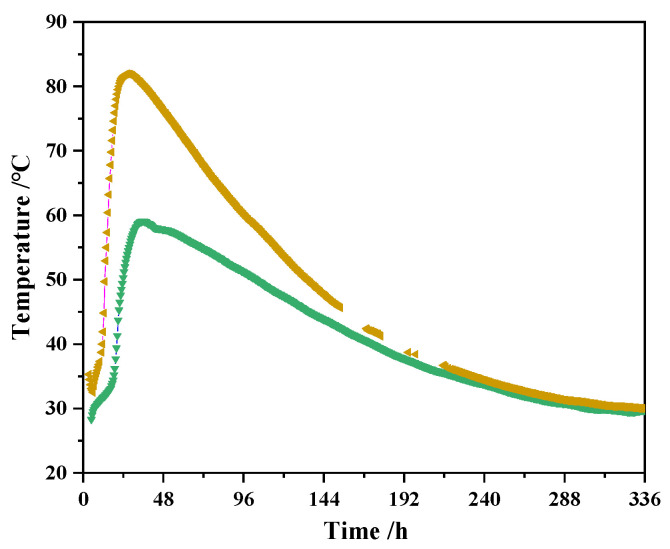
Temperature variation process of mass concrete measured.

**Figure 2:**
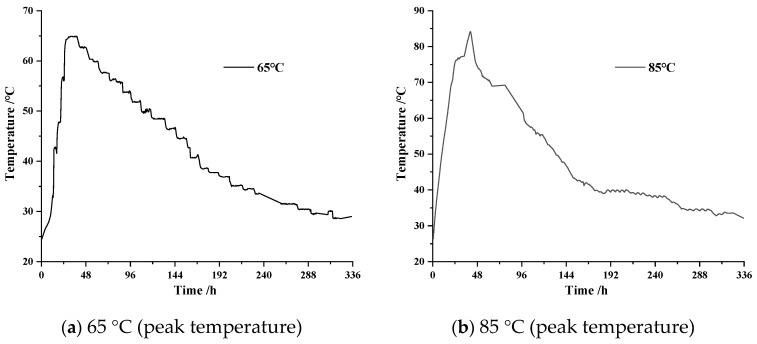
Variable temperature curing environment simulated by curing box: (**a**) 65 °C (peak temperature); (**b**) 85 °C (peak temperature).

The temperature simulation process was as follows: the temperature change process of concrete was broken down into several small temperature change stages. Additionally, the temperature was adjusted every 4 h during the heating phase until it increased to the maximum temperature; the temperature was adjusted every 8 h during the cooling phase until it dropped to an ambient temperature. The heating and cooling rate of the temperature was determined according to the concrete temperature data measured in the engineering. Figure 1 shows the temperature variation process inside two groups of concrete walls that were measured in a C50 mass concrete engineering project. As can be seen from Figure 1, the internal temperature of concrete reached its maximum value around 24–48 h (taking its time when concrete began to be poured as the initial zero point), and the internal temperature of concrete was basically consistent with the ambient temperature at 14 d, i.e., the cooling process was basically over. The simulation of the temperature variation process in this study was based on the temperature variation in the data of mass concrete obtained from engineering practice (as shown in Figure 1), and Figure 2 shows the variable temperature curing environment with peak temperatures at 65 °C and 85 °C, which were simulated by a curing box based on the temperature change data obtained in engineering practice.

#### 2.2.2. Preparation and Curing of Cement Pastes

The tests involved a total of two cementitious systems, and the raw material formulation of the formed cement paste is shown in Table 2. The MEA content in the two cementitious systems accounted for 8% of the total mass of the cementitious materials. The cement paste test block used to test the hydration of MgO was molded in Φ25 mm × 30 mm columnar rigid PVC molds with a water-cement ratio of 0.32. Before forming, the raw materials were evenly mixed by a mixer, and the evenly mixed cement paste was put into the test mold by a cement paste purifying mixer with water for mixing. Then, the test mold was placed on a shaking table for 60 s to eliminate the air inside the paste. The prepared cement paste specimens were maintained in the curing environment shown in Figure 2 to observe the hydration of MgO in the cement paste.

The experiment involved two curing methods: water curing and non-wet curing. The prepared cement paste specimens were cured directly with a mold (the mold could be sealed with a cover), and the mold was wrapped with plastic wrap to isolate water so as to realize the not-wet curing. The specimens of the cement paste cured in water were removed from the mold and placed in water for curing after 20 h of curing with the mold. In addition, paste specimens with curing ages of 1 d, 3 d, 7 d and 14 d were selected to complete the characterization of MgO’s hydration degree under the whole temperature course (take the preparation time of ready cement paste as the initial zero point).

#### 2.2.3. Determination of the Remaining MgO Content in Cement Paste

The K-value method (XRD) was used to determine the content of MgO in the cement paste for hydration. ZnO was chosen as the internal standard substance (the dosage is 2 wt.%). The scanning range was 35°–45°, and the scanning speed was 1°/min. ZnO and MgO powders were, respectively, weighed according to the mass ratio of 1:1, and the powders were ground with an agate mortar to obtain a homogeneous mixed powder sample. The milled powder sample was subjected to X-ray diffraction analysis, and the integrated area of the strongest diffraction peaks of ZnO and MgO in the diffraction pattern was calculated using MDI Jade software. The ratio of the integrated area of the strongest diffraction peaks of ZnO and MgO was the K value, which was calculated to be 0.57. The position of the strongest diffraction peak of ZnO was d_101_ = 2.47, and the position of the strongest diffraction peak of MgO was d_200_ = 2.11. The remaining content of MgO in the cement paste was calculated by Equation (1):

(1) $$\omega_{\mathrm{MgO}}=K\;\frac{(1-\omega_{\mathrm{ZnO}})}{\omega_{\mathrm{ZnO}}}\;\frac{I_{\mathrm{ZnO}}}{I_{\mathrm{MgO}}}$$

where ω_MgO_ is the remaining content of MgO in the cement paste. ω_ZnO_ is the doping amount of the internal standard substance ZnO. I_ZnO_ is the integrated intensity of the strongest diffraction peak of the internal standard substance ZnO, and I_MgO_ is the integrated intensity of the strongest diffraction peak of MgO. K is the characteristic constant (K = 0.57).

## 3. Results and Discussion

### 3.1. Effect of Curing Temperature on MgO Hydration in Cement Paste

**Figure 3:**
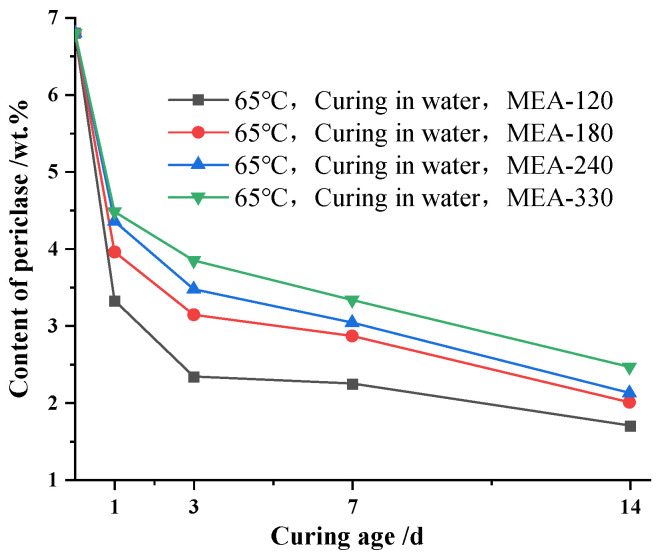
Content of periclase for different ages in cement pastes with MEA cured in water at 65 °C variable temperature condition.

In this section, the effect of the curing temperature on MgO hydration in cement paste was studied. The cement paste was prepared according to the raw material ratio of “system a” in Table 2. Figure 3 shows the hydration process of MgO when mixed with four types of MEA cement paste under a 65 °C variable temperature with water curing conditions. It can be seen from Figure 3 that the changes in the MgO content in the cement paste mixed with four types of MEA generally showed a trend of first fast and then slow. The hydration rate of MgO in the cement paste was the fastest before 1 d, and then the hydration rate of MgO in the cement paste began to gradually slow down. When the curing age was 1 d, the content of MgO in the cement paste mixed with MEA-120, MEA-180, MEA-240 and MEA-330 was reduced by 3.48 wt.%, 2.85 wt.%, 2.45 wt.% and 2.33 wt.%, respectively. In contrast, the content of MgO in the cement paste mixed with MEA-120, MEA-180, MEA-240 and MEA-330 was reduced by 0.99 wt.%, 0.81 wt.%, 0.88 wt.% and 0.63 wt.% at the curing age from 1 d to 3 d, respectively. In addition, the higher the activity of MEA, the faster the hydration at the early stage, and the lower MgO remained in the cement paste during the cooling stage. The cement pastes with MEA-120 had the lowest residual MgO content throughout the process, while the cement paste with MEA-330 had the highest residual MgO content throughout the process. The residual MgO content of the cement paste with the other two activities always lay between them.

**Figure 4:**
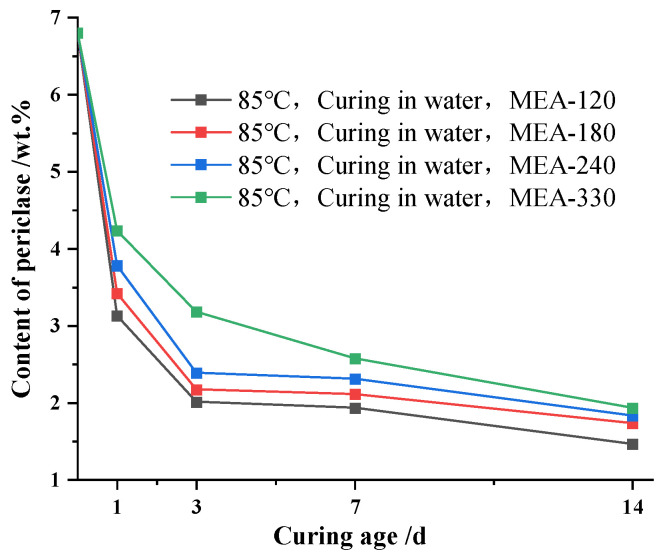
Content of periclase for different ages in cement pastes with MEA cured in water at 85 °C with variable temperature conditions.

Figure 4 shows the hydration process of MgO mixed with four MEA cement pastes under 85 °C variable temperature water curing conditions. As can be seen from Figure 4, the trend in the change in the MgO content in cement paste mixed with four kinds of MEA at 65 °C and 85 °C and under a variable temperature water curing condition was basically similar. The hydration rate of MgO in the cement paste was the fastest at 1 d, and then the hydration rate started to decrease gradually. However, compared to the 65 °C variable temperature water curing condition, the residual content of MgO in the cement paste under an 85 °C variable temperature water curing condition at a curing age of 1 d was significantly reduced. Moreover, the reaction rate of MgO in the cement paste became slower during the cooling stage, and the content of MgO in the cement paste of multiple MEA-doped groups appeared as a “plateau period”., i.e., the content of MgO remained essentially unchanged.

**Figure 5:**
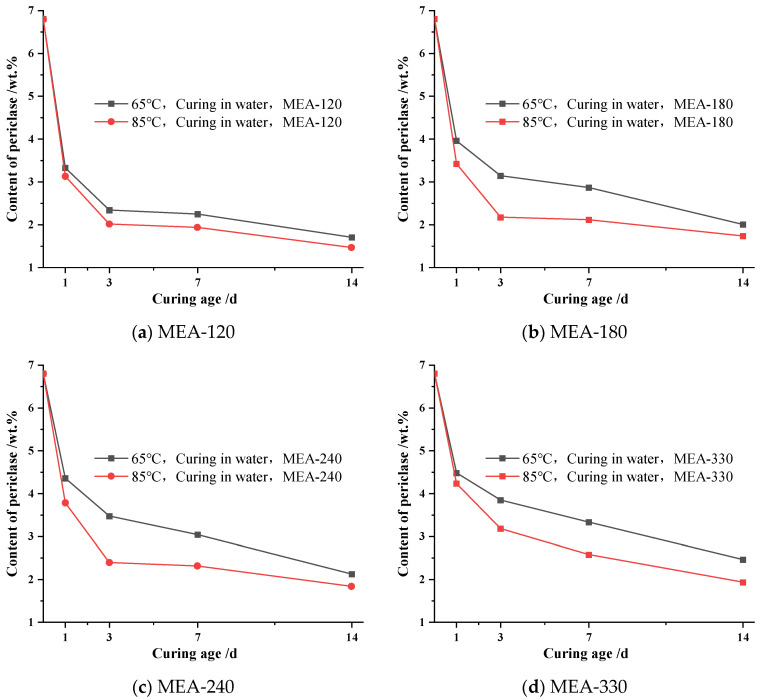
Content of periclase for different ages in cement pastes mixed with the same MEA cured in water at 65 °C, 85 °C variable temperature: (**a**) MEA-120; (**b**) MEA-180; (**c**) MEA-240; (**d**) MEA-330.

Figure 5 shows the comparison of the remaining MgO content of the cement paste mixed with the same active MEA at 65 °C and 85 °C under variable temperature water curing conditions. By comparison, it was found that the residual content of MgO in the cement paste mixed with MEA-120, MEA-180, MEA-240 and MEA-330 at the curing age of 1 d under an 85 °C variable temperature and the water curing condition is reduced by 0.2 wt.%, 0.54 wt.%, 0.57 wt.% and 0.24 wt.%, respectively, compared with that under a 65 °C variable temperature water curing condition. Moreover, the MgO content in the cement paste mixed with MEA-120 under a 65 °C variable temperature water curing condition and MEA-120, MEA-180 and MEA-240 under an 85 °C variable temperature water curing condition showed a “plateau period” during the cooling stage (3–14 d). This was not significantly observed for other cement pastes when mixed with other active MEAs under the same curing condition. In comparison, it was found that the MgO content in the cement paste of these four groups started to show signs of a “plateau” mostly at the age of 3 d (i.e., the cooling stage). At this time, the MgO content in the cement paste was mixed with MEA-120 under a 65 °C variable temperature water curing condition, MEA-120, MEA-180 and MEA-240 was under an 85 °C variable temperature water curing condition and were 2.33 wt.%, 2.01 wt.%, 2.17 wt.% and 2.38 wt.%, respectively. The other four groups of cement pastes without this situation had a higher MgO content at a curing age of 3 d compared to the MgO content values at the time of plateauing. In addition, it could also be found that the increase in the curing temperature had a slightly different effect on the promotion of the hydration reaction of different active MEAs. The low activity of MEA seemed to be more sensitive to the temperature, and the increase in the temperature was more effective when promoting the hydration of low-activity MEA. This may also be due to the rapid reaction of the highly reactive MEA, resulting in a premature approach to the “plateau content” of MgO in the cement paste mixed with a highly reactive MEA.

### 3.2. Effect of Curing Methods on the Hydration of MgO in Cement Paste

**Figure 6:**
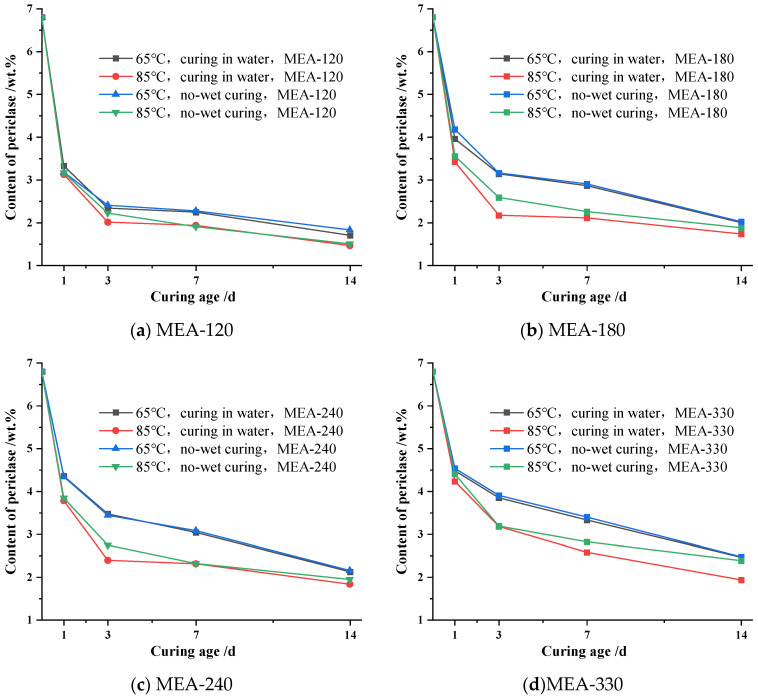
Content of periclase for different ages in cement pastes mixed with the same MEA under different curing conditions: (**a**) MEA-120; (**b**) MEA-180; (**c**) MEA-240; (**d**) MEA-330.

In order to clarify the effect of water curing and non-wet curing on MgO hydration in the cement paste under variable temperature conditions, the hydration of MgO in the cement paste was compared under two curing methods according to “system a” forming cement paste. Figure 6 shows the comparison of MgO residual content in cement paste mixed with the same active MEA by different curing methods under variable temperature conditions of 65 °C and 85 °C.

As can be seen in Figure 6, for cement paste mixed with the same active MEA curing in the same variable temperature environment, the effect of non-wet curing and water curing on the hydration of MgO in the cement paste was not as great as the effect of temperature on the hydration of MgO. The effect of moisture on MgO hydration in the MEA mixed cement paste was mainly reflected in the period before the curing age of 3 d, and the promotion effect of moisture on MgO hydration in the cement paste in the subsequent process seemed not to be not obvious. It can be seen from the changing curve of MgO content in the cement paste underwater with non-wet curing at a 65 °C variable temperature that the difference between non-wet and water curing methods on MgO hydration was very small at the curing age of 1–3 d, and the remaining MgO content in the cement paste under the two curing methods of MEA-120, MEA-180, MEA-240 and MEA-330 was basically the same. In addition, in the subsequent cooling process, no significant difference was observed in the content of MgO on the paste under two curing conditions, even with the different curing methods. As can be seen from the variation curves of the content of MgO in cement paste under 85 °C variable temperature conditions for water and non-wet curing, compared with non-wet curing, the remaining content of MgO in the cement paste mixed with MEA under water curing significantly decreased at this stage before the curing age of 3 d, where no significant difference was found in the change in the MgO content in the paste under the two curing methods of water and non-wet curing in the cooling stage after 3 d.

When comparing the hydration process of MgO in the cement paste in Figure 6c, it was found that the content of MgO in the cement paste mixed with MEA was significantly reduced when the temperature peak of variable temperature curing increased from 65 °C to 85 °C under the same non-wet condition. Compared to 65 °C variable temperature non-wet curing, the residual content of MgO in the cement paste at 85 °C variable temperature non-wet curing ages of 1 d, 3 d, 7 d and 14 d decreased by 0.50 wt.%, 0.69 wt.%, 0.76 wt.% and 0.21 wt.%, respectively. However, under the same 65 °C variable temperature curing condition, when the curing method changed from non-wet curing to water curing, there was no significant difference in the content of MgO in the cement paste when mixed with MEA-240 during the whole age. In addition, it could also be found that the MgO content in the cement paste mixed with MEA-240 was basically the same in this stage before the maintenance age of 3 d under the condition of water curing and non-wet curing at a 65 °C variable temperature curing. However, when the temperature peak of variable temperature curing from 65 °C increased to 85 °C, compared with the non-wet curing, the content of MgO in the water-curing cement paste mixed with MEA-240 decreased significantly by 0.35 wt.% at the curing age of 3 d. The variation in the MgO content in the cement paste when mixed with other active MEA in Figure 6 was also similar to the results shown in Figure 6c.

### 3.3. Effect of Cementitious System on MgO in Cement Paste

**Figure 7:**
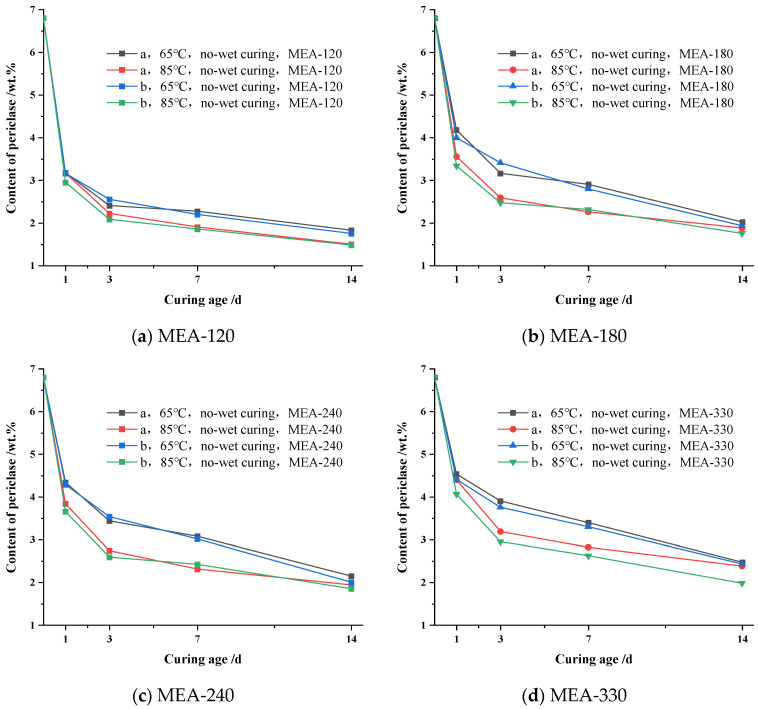
Content of periclase at different ages in cement pastes prepared by Cementitious system a and system b cured in non-wet conditions at different variable temperatures: (**a**) MEA-120; (**b**) MEA-180; (**c**) MEA-240; (**d**) MEA-330.

In order to clarify the effect of the cementitious system on MgO hydration in the cement paste under variable temperatures and non-wet curing conditions, the hydration of MgO in the cement paste was formed according to the cementitious system a and b was compared. Figure 7 shows the comparison of the MgO residual content in cement paste mixed with the same active MEA under the condition of variable temperature and non-wet curing at 65 °C and 85 °C.

As can be seen from Figure 7, for the cement paste formed according to the cementing systems a and b and mixed with the same active MEA, there was little difference in the variation in the MgO content in the cement paste under the same curing condition. The content of MgO in cement paste formed according to cementing system b was lower than that of the cement paste formed according to system a at all ages. This may be caused by the addition of an 8 wt.% S95 slag powder in system a to replace part of the cement without changing the yield of fly ash. According to Figure 7d, under variable temperature and non-wet curing at 65 °C, the residual content of MgO in the cement paste was formed according to the cementitious system a and was 0.12 wt.%, 0.14 wt.%, 0.10 wt.% and 0.04 wt.% higher than that in the cementitious system b at the age of 1 d, 3 d, 7 d and 14 d, respectively. Therefore, it can be considered that the addition of the 8 wt.% slag powder with the same fly ash content could inhibit the hydration of MgO in the cement paste; however, the inhibition effect was not obvious.

In addition, by comparing MgO hydration in the cement paste after the cementitious system and temperature change in Figure 7, it was found that the effect of temperature variation on MgO hydration in the cement paste was obviously greater than that of the cementitious system., i.e., the temperature was still the main factor affecting MgO hydration in cement paste.

## 4. Conclusions

1. Under variable temperature curing conditions with a peak temperature of 65 °C and 85 °C, the hydration of MgO in cement paste presented a trend that produced first fast and then slowly changed. The MgO of the cement paste mixed with highly active MEA was nearly half hydrated at the curing age of 1 d. After that, the change in the remaining content of MgO in the cement slurry slowed down significantly.
2. When the peak temperature of variable temperature curing increased from 65 °C to 85 °C, it accelerated the hydration rate of MgO in the cement paste in the early stage (1–3 d), and the increase in the curing temperature led to a plateau period for the change in the MgO content in cement paste. It was observed that when there was a plateau of the MgO content change, MgO in the cement paste was nearly 70% hydrated (remaining content above 2.0 wt.%).
3. When the curing temperature and the cementitious system were the same, the effect of curing methods on the hydration of MgO in the cement paste was mainly reflected in the early stage (1–3 d). With the increase in the curing age, the difference in the MgO content caused by curing methods gradually decreased. In addition, the increase in the variable curing temperature could aggravate the effect of curing methods on MgO hydration in the cement paste.
4. By comparing the effects of temperature, the curing methods and the cementitious system on MgO hydration in the cement paste, it was found that temperature was the main factor affecting the hydration of MgO. The increase in the temperature could significantly promote the hydration of MgO in cement paste, under the condition of non-wet curing, compared to 65 °C variable temperature non-wet curing. The residual content of MgO in cement paste at a 85 °C variable temperature with non-wet curing ages 1 d, 3 d, 7 d and 14 d decreased by 0.50 wt.%, 0.69 wt.%, 0.76 wt.% and 0.21 wt.%, respectively; the other two had effects on the hydration of MgO, but this effect was not obvious.

## Figures and Tables

**Table 1:** Chemical compositions of raw materials.

Item	Chemical Components (%)								
	CaO	SiO_2_	Al_2_O_3_	Fe_2_O_3_	MgO	SO_3_	Na_2_O	K_2_O	LOSS
Portland cement	65.32	18.55	3.95	3.41	1.01	2.78	0.72	0.17	2.88
Fly ash	4.07	50.53	31.65	4.48	0.91	1.32	0.67	1.26	2.77
Slag	38.00	33.72	17.74	0.77	6.35	1.04	0.41	0.40	−0.72
MEA-120	1.89	3.38	0.54	0.66	85.23	0.92	-	-	3.12
MEA-180	3.90	5.17	0.64	0.68	85.57	0.75	-	-	2.97
MEA-240	1.88	4.07	0.85	0.78	90.45	0.03	-	-	1.53
MEA-330	4.37	5.82	0.71	0.61	85.71	0.60	-	-	1.88

**Table 2:** Mix proportion of cement paste/g.

System	Cement	Fly Ash	Slag	MEA	Water
a	66	18	8	8	32
b	74	18	-	8	32

## Data Availability

The data presented in this study are available on request from the corresponding author.
